# Ketamine-assisted psychotherapy treatment of chronic pain and comorbid depression: a pilot study of two approaches

**DOI:** 10.3389/fpain.2023.1127863

**Published:** 2023-05-19

**Authors:** Daniella Batievsky, Michelle Weiner, Shari B. Kaplan, Michael Edward Thase, Domenick Nicholas Maglione, Denise Christina Vidot

**Affiliations:** ^1^ Department of Psychology, University of Pennsylvania, Philadelphia, PA, United States; ^2^ Spine and Wellness Centers of America, Miami, FL, United States; ^3^ Cannectd Wellness, Boca Raton, FL, United States; ^4^ Department of Psychiatry, Perelman School of Medicine, Philadelphia, PA, United States; ^5^ Corporal Michael J. Crescenz VA Medical Center, United States Department of Veterans Affairs, Philadelphia, PA, United States; ^6^ School of Nursing and Health Studies, University of Miami, Coral Gables, FL, United States

**Keywords:** ketamine, depression, pain, intramuscular, therapy, microdose, psychedelic, psycholytic

## Abstract

Chronic pain and depression diagnoses are skyrocketing. There is an urgent need for more effective treatments. Ketamine was recently established to alleviate pain and depression, but many gaps remain in the scientific literature. This paper reports the findings of an observational preliminary study that explored the efficacy of ketamine-assisted psychotherapy (KAPT) for chronic pain/major depressive disorder (MDD) comorbidity. Researchers evaluated two KAPT approaches to determine optimal route of administration/dose. Ten individuals diagnosed with a chronic pain disorder and MDD receiving KAPT were recruited: five individuals pursuing the psychedelic approach (high doses administered intramuscularly 24 h before therapy) and five individuals pursuing the psycholytic approach (low doses administered sublingually via oral lozenges during therapy). To evaluate differences between altered states of consciousness each approach induces, participants completed the Mystical Experience Questionnaire (MEQ30) after their first (T-1), third (T-2) and sixth/final (T-3) treatment sessions. Primary outcomes were change in Beck Depression Inventory (BDI) scores and Brief Pain Inventory (BPI) Short Form scores from baseline (T0) to (T-1)–(T-3). Secondary outcomes were changes in Generalized Anxiety Disorder (GAD-7) Scale scores and Post-Traumatic Stress Disorder Checklist (PCL-5) scores at each timepoint. Statistically significant differences between each approach were not observed, but the small sample’s limited statistical power makes changes seen worth noting. All participants’ symptoms declined throughout treatment. Psychedelic treatment participants saw a larger, more consistent decrease. Researchers conclude that KAPT may be effective for treating chronic pain/MDD comorbidity, anxiety and Post-Traumatic Stress Disorder (PTSD). Findings imply that the psychedelic approach may be more effective. This pilot study serves as a basis for more extensive research that will inform how clinicians administer treatment to optimize outcomes.

## 1. Introduction

The mental health and chronic pain crisis in the US have grown to epidemic proportions. As mental illness grows, limited efficacy of mainstream treatment modalities leaves many searching for alternative therapies. Depression is the leading cause of disability in the US, yet it is becoming increasingly resistant to medication and therapy can take years to decades to achieve significant results (1). Ironically, there is a positive correlation between the increase in depressive disorder diagnoses and the growth of pharmaceutical prescriptions (2). In 2008, Medicaid funded $3.6 billion for psychiatric drugs and in 2013, one in six Americans reported taking at least one psychiatric drug to treat depression and/or anxiety. Yet, one in three patients who take antidepressants to treat major depressive disorder (MDD) do not see any improvement (3–5). Between 2010 and 2018, the cost of MDD in the US increased from $236.6 billion to $326.2 billion (6).

At the same time that depression diagnoses are skyrocketing, the number of individuals living with chronic pain conditions are also growing. According to the National Institute of Health (NIH), the number of US adults “*suffering from at least one painful health condition increased substantially from 120.2 million (32.9%) in 1997/1998 to 178 million (41%) in 2013/2014”* (7). Similarly to depression, pharmaceutical prescription rates are increasing at the same time that individuals living with chronic pain conditions are increasing. Opioid prescription rates more than doubled between 2001/2002 (4.1 million) and 2013/2014 (10.5 million) and quadrupled between 1999 and 2010 (8). About $560 billion is lost each year due to the resulting medical costs, disability programs and lost productivity (7, 9). Thus, there is a critical need for more effective treatment options to better aid the increasing numbers of people who are suffering from both depressive disorders and chronic pain conditions.

Ketamine, primarily known as the most widely used anesthetic in the world, has recently been established to effectively treat depressive and chronic pain disorders (10–13). The treatment has a variety of physiological mechanisms of action. As an N-methyl-D-aspartate (NMDA) receptor antagonist, ketamine blocks NMDA receptor pathways, which enhances glutamatergic activity and increases *α*-amino-3-hydroxy-5-methyl-4-isoxazolepropionic acid (AMPA) receptor signaling. AMPA stimulation increases brain-derived neurotrophic factor (BDNF), which stimulates synaptogenesis, or the formation of new receptors and synapses. The process of creating connections between neurons is often completely compromised for individuals suffering from depression, so the increased synaptogenesis and neuroplasticity works to promote restoration of those lost neural connections and reverse any stress-induced neuronal changes (14). NMDA receptor activation is also a core cause of central sensitization (the increased responsiveness of nociceptive neurons to normal stimuli), which leads to wind-up (the persistent up-regulation and high reactivity of the nervous system). Thus, ketamine may play a major role in reducing the central sensitization and wind-up that is seen in individuals suffering from depression and chronic pain (15). NMDA receptors are not only implicated in the pathophysiology of depression, but are also implicated in the etiology and maintenance of chronic pain disorders; ketamine's action as an NMDA receptor antagonist makes it an ideal treatment option (13). Ketamine's role in cortical disinhibition may also contribute to antidepressant responses; ketamine strengthens gamma band electroencephalography power, which causes limbic cortical disinhibition, and thereby increases the availability of dopamine in the brain (14, 16). In addition, ketamine reduces inflammation signaling, which is elevated in those diagnosed with MDD and chronic pain disorders (17). Ketamine also has psychological mechanisms of action. Individuals undergoing treatment report that they finally feel safe enough to face past trauma, work through uncomfortable emotional blockages, and gain insight regarding the root causes of their depression and/or pain. Individuals commonly experience an increased sense of unity, interconnectedness, spirituality, and meaning during treatment, which is attributed to its rapid healing effects (14, 18–20).

As a result of the promising findings from the scientific literature thus far, Spravato®, a nasal spray that delivers the S-enantiomer of ketamine, was approved for treatment-resistant depression in 2019 (5). Although other forms of ketamine are only FDA approved for surgical and anesthetic indications, clinicians have become more open to administering off-label ketamine (orally, intramuscularly, intravenously, and sublingually) to successfully treat depression and chronic pain conditions. As interest in ketamine has grown, more and more ketamine clinics continue to open their doors.

Because the adoption of ketamine as a viable treatment option has been so recent, many gaps exist in the current ketamine literature (5). First, ketamine's optimal route of administration remains unclear. There is sufficient data to support ketamine's efficacy for depression and pain indications when administered intravenously, but there is limited data that sufficiently investigates intramuscular (IM), sublingual, and oral routes of administration (21, 22). More high quality, quantitative research is needed to sufficiently assess intramuscular, sublingual, and oral routes of ketamine administration to determine which is optimal.

Second, the optimal dose of ketamine warrants further study. Because there is limited research that investigates whether an altered state of consciousness mediates ketamine's therapeutic effect, clinicians are unclear as to whether they need to administer a dose high enough to induce one (23). Many clinicians believe that ketamine's primary mechanisms of action are its ample physiological and neurobiological effects, so they argue that high doses are not essential to achieve symptom relief (24). On the other hand, many clinicians argue that high doses are critical because ketamine's primary mechanism of action is the psychological insight that stems from altered states of consciousness and “mystical experiences” (11, 15). Due to clinicians’ differing opinions, the existing conflicting research, and the lack of ample data, researchers must conduct more studies that adequately compare different doses of ketamine and the altered states of consciousness they induce (11, 18, 24). It is essential to discover whether ketamine has a dose-dependent effect to determine its ideal dose.

The therapeutic potential of ketamine may be particularly relevant if a connection between trauma, chronic pain, and depression is made. However, there is minimal understanding of the role that emotional trauma plays in the development and maintenance of chronic pain disorders. The etiology of chronic pain disorders is mainly believed to be solely physiological, but potential psychological mechanisms that may contribute to the experience of chronic pain remain largely unexplored. Central sensitization (CS) is associated with the development and persistence of chronic pain conditions and occurs when neural signaling in the central nervous system (CNS) has been amplified through the wind-up process, and gets regulated into a state of constant high reactivity (15). Trauma and poor mental health have been shown to cause CS and put individuals in that constant state of reactivity associated with chronic pain. For example*, “rates of psychosocial trauma and lifetime adversity are substantially elevated in patients with pain disorders, with PTSD prevalence estimated at 20.5% in patients with chronic widespread pain, and those with a trauma history being approximately three times more likely to develop pain conditions involving CS later in life than those without a trauma history. Individuals with trauma histories tend to have worse pain and health outcomes, including more severe symptom presentation, increased disability, increased likelihood of unemployment, and higher healthcare utilization”* (25). Thus, underlying emotional trauma, anxiety, or depression may contribute to central sensitization and ultimately, the experience of chronic pain. For example, a randomized controlled trial that investigated the efficacy of IV ketamine for complex regional pain syndrome (CRPS) found that participants in the IV ketamine group had significant reductions in pain symptoms, whereas participants in the placebo group had no reductions in pain symptoms according to multiple pain parameters; ketamine is therefore effective for the CRPS patient population.

Because CRPS typically develops after the experience of a traumatic event, trauma may play a role in the etiology and maintenance of the chronic pain disorder that results. Ketamine's ability to significantly reduce symptomatology may indicate a different mechanism of action; in addition to its neurobiological mechanisms, ketamine may work psychologically to help individuals cope with their trauma. As a result of reducing their emotional pain, ketamine may reduce their experience of physical pain (26). Since many people who live with chronic pain conditions also experience trauma and depression, whether trauma and underlying mental health issues contribute to the rise of chronic pain conditions is an important question that needs to be further explored. It is essential to fully understand how chronic pain conditions develop and function to advance treatments that ensure maximum pain relief.

Fourth, further research is needed to determine if the effects of ketamine can be enhanced or extended by pairing the treatment with evidence based psychological interventions. The majority of the existing literature assesses ketamine's efficacy without therapy (10–12, 24, 26). According to the “process of change” model, however, when psychedelics are administered alongside therapy, a supportive social and environmental context, mental and neuronal plasticity is enhanced and patients are further able to reprocess, adapt, and change. The combination of increased plasticity and psychotherapeutic support leads to psychologically and cognitively flexible states that promote relearning and acceptance (18, 27, 28). Further research is necessary to test the “process-of-change” model and analyze the efficacy of ketamine-assisted psychotherapy to determine whether therapy enhances therapeutic outcomes.

Lastly, although research has evaluated ketamine's efficacy for the treatment of depression and chronic pain indications separately, there is minimal research that evaluates ketamine's efficacy for the treatment of depression comorbid with a chronic pain disorder. It is extremely common for individuals suffering from chronic pain conditions to also suffer from anxiety, depression, and/or PTSD. According to recent research*, “Depression and chronic pain frequently coexist, with up to 60% of chronic pain patients also presenting with depression. Furthermore, the combination of chronic pain and depression leads to poorer treatment outcomes [with antidepressants] and overall functioning than either condition alone”* (29). Given their high comorbidity and the limited efficacy of the currently available treatment options, there is an urgency for researchers to examine ketamine's utility for individuals diagnosed with both depression and a chronic pain disorder.

In sum, many questions essential to maximizing ketamine treatment remain unanswered (14). There is limited research examining ketamine's efficacy administered via intramuscular, oral and sublingual routes, effectiveness alongside therapy, and antidepressant and pain alleviating effects specifically on chronic pain syndromes with comorbid depression (22, 24). Clinicians do not know whether mystical experiences/dissociations are key to healing, so ketamine's optimal dose remains unclear (21). Furthermore, despite the potent findings of the research to date, researchers across most studies acknowledged their results' severe limitations due to small sample sizes and low-quality study designs (10–12, 22).

This study aims to fill the gaps in the current ketamine literature to provide clinicians with greater knowledge regarding (1) ketamine-assisted psychotherapy's efficacy for the specific population of patients diagnosed with both a chronic pain disorder and a depressive disorder, (2) ketamine's optimal route of administration, (3) ketamine's optimal dose, and (4) the connection between trauma, chronic pain, and depression. It is critical to acquire the answers to these lingering questions to understand how to best administer ketamine treatment to optimize outcomes.

Researchers pose the following questions: What are the impacts of two different ketamine-assisted psychotherapy (KAPT) approaches on chronic pain patients diagnosed with a comorbid depressive disorder? Does ketamine have a superior route of administration? Does ketamine have a dose-dependent effect on symptom reduction (i.e., are greater altered states of consciousness critical to symptom relief)?

This study adds to ketamine's research base by analyzing the survey outcomes of patients diagnosed with major depressive disorder (MDD) and a comorbid chronic pain disorder receiving two different approaches to ketamine-assisted psychotherapy (KAPT): the psychedelic approach vs. the psycholytic approach. Throughout this paper, the *psychedelic* approach refers to high dose, intramuscular ketamine injections that are followed by therapy sessions 24 h later. The *psycholytic* approach refers to low doses of ketamine delivered sublingually via oral lozenges during therapy sessions.

Principal findings support KAPT's efficacy for the treatment of depression and chronic pain comorbidity and indicate its potential use for anxiety and PTSD indications. Results suggest that the psychedelic approach may be superior to the psycholytic approach, which implies that larger doses and intramuscular routes may be optimal. Further research is necessary to further explore and support these claims.

## 2. Methods

### 2.1. Participants

Ten (*N* = 10) adults with a prior diagnosis of major depressive disorder (MDD) and a comorbid chronic pain condition were selected to participate in the study. Researchers identified and recruited participants by administering flyers to patients of Spine and Wellness Centers of America and Cannectd Wellness in person and online via website and social media strategies. A screening survey was administered to prospective participants to ensure that they qualified for the study according to the following criteria:

#### 2.1.1. Inclusion criteria

• Adults 25 years old or older
• Prior diagnosis of a chronic pain disorder (lasting more than 6 months)
• Prior diagnosis of major depressive disorder (MDD)

#### 2.1.2. Exclusion criteria

• Pregnant women
• Diagnosis of schizophrenia or psychosis
• Suicidal ideations
• Cardiac event in the past 6 months
• Uncontrolled hypertension
• Adults unable to provide informed consent
• Prisoners

### 2.2. Procedures involved

Participants were placed into one of the two treatment groups based on the recommendations of their integrative pain management physician, Dr. Michelle Weiner, and their ketamine-assisted psychotherapist, Shari Kaplan, LCSW. Recommendations were based on weight, previous psychedelic use, levels of anxiety regarding entering non-ordinary states of consciousness (NOSC), and treatment intention. Five (*n* = 5) participants were placed into the psychedelic group and five (*n* = 5) participants were placed into the psycholytic group.

#### 2.2.1. Therapy

A trained integrative mental health clinician met with each participant prior to their first ketamine treatment for a thorough, background therapy session. The different aspects of the therapy assessment were as follows: reviewing trauma, reviewing birth, discussing early childhood and adolescence history, reviewing upbringing, conducting mental health and psychosocial assessments, discussing intentions and goal setting, discussing the post-session integration process. A workbook was given to the participants to guide them through what to expect from ketamine treatment.

In their subsequent six sessions, the therapist facilitated dynamic, intensive, collaborative and strength/resiliency enhancement focused therapy using eye movement desensitization and reprocessing (EMDR). Participants learned self-regulation skills, meditation, and mindfulness practices.

Participants who were treated with a psychedelic dose of ketamine completed therapy sessions 24 h after ketamine administration, while participants who were treated with a psycholytic dose of ketamine actively participated in a therapy session immediately upon administration.

#### 2.2.2. Psychedelic intramuscular ketamine injections

Over a series of six separate sessions, five participants (*n* = 5) were treated with intramuscular ketamine injections, with dosages ranging from 40 to 100 mg. The participants underwent 6 sessions in 6 weeks, receiving two IM injections per session. The standard IM injection dosage regimen is as follows: first session—40 and 50 mg, second session—50 and 60 mg, third session—60 and 70 mg, fourth session—70 and 80 mg, fifth session—80 and 90 mg, sixth session—90 and 100 mg.

To enable administration of higher doses at session six, participants were treated with an escalation of dose protocol: they were administered half the dose they received during session six at session one, which increased by 10 mg at each consecutive session.

#### 2.2.3. Psycholytic oral/sublingual ketamine lozenges

Over a series of six separate sessions, five participants (*n* = 5) were treated with oral ketamine lozenges, with dosages ranging from 25 to 75 mg. The participants underwent 6 sessions in 6 weeks. The standard oral dosage regimen is as follows: first and second session—25 mg, third and fourth session—50 mg, fifth and sixth session—75 mg. Upon ingestion, participants were told to hold and swish the contents of the lozenge in their mouths for 15 min to maximize contact with the mucosal surface. Thus, this route of administration was both oral and sublingual.

Since participants actively participated in a therapy session immediately upon ketamine administration, participants were treated with an escalation of dose protocol within a range that allowed them to effectively communicate and process with their therapist.

#### 2.2.4. Outcome measures

Participants completed a variety of surveys at multiple timepoints throughout their course of treatment. The Beck Depression Inventory (BDI) and the Brief Pain Inventory (BPI) Short Form were utilized to determine each treatment's impact on depressive and pain symptoms. Because participants included in this study also had anxiety and thorough trauma histories, the Generalized Anxiety Disorder (GAD-7) Scale was utilized to determine ketamine's impact on participants' anxiety symptoms and the Post-Traumatic Stress Disorder Checklist (PCL-5) was utilized to measure ketamine's impact on PTSD severity.

The Mystical Experience Questionnaire (MEQ30) was utilized to measure ketamine's consciousness altering effects. Although not the primary outcome measure of this study, the MEQ30 is discussed first in the results and discussion sections. It is necessary to first determine whether each treatment approach produced different altered states of consciousness; the remainder of the outcome measures are utilized to determine whether any differences observed produced distinct impacts on participants' symptoms.

##### 2.2.4.1. Beck Depression Inventory (BDI)

1. A 21-item self-report rating inventory that measures characteristics, attitudes, and symptoms of depression (30)

##### 2.2.4.2. Brief Pain Inventory (BPI) short form

2. A 9 item self-report questionnaire that measures severity of pain and impact of pain on daily functioning (31)

##### 2.2.4.3. Generalized Anxiety Disorder (GAD-7) scale

3. A seven-item, self-report scale designed to screen, diagnose and measure the severity of anxiety disorder (32)

##### 2.2.4.4. Post-Traumatic Stress Disorder Checklist (PCL-5)

4. A 20 item self-report survey that evaluates all the PTSD symptoms in the DSM-5 (33)

##### 2.2.4.5. Mystical Experience Questionnaire (MEQ30)

5. A self-report questionnaire used to measure mystical-type experiences in studies where hallucinogens are used (34)

Participants completed the first 4 scales before they received any treatment (baseline; time point 0- T-0). Scale number 5 was not administered to participants at baseline (T-0) because responses are dependent upon the experience of the treatment itself. All 5 scales were then administered after the first treatment session (T-1), after the third treatment session (T-2), and after the sixth treatment session (T-3). Clinicians measured each participant's blood pressure and pulse before and after each treatment session.

### 2.3. Data analysis

This study follows a single-subject experimental design, where the repeated measurements aid in facilitating a better understanding of the individual's variability in response to treatment. Because questionnaires were administered prior to receiving any treatment, the participants acted as their own control, where their T-1–T-3 ratings were compared against their baseline T-0 ratings. Consequently, this methodology allowed researchers to determine the short-term effects of ketamine-assisted psychotherapy treatment on participants' pain, depression, anxiety, and PTSD symptoms.

Researchers also compared outcome measures of the participants who received psychedelic treatment to the outcome measures of the participants who received psycholytic treatment at all timepoints. A chi-squared test was conducted for each treatment group's mean outcome measure scores at timepoints (T-1)–(T-3). With this methodology, researchers compared the effectiveness of two different approaches to ketamine-assisted psychotherapy treatment: larger doses administered intramuscularly 24 h prior to therapy vs. smaller dosages administered orally/sublingually during therapy.

### 2.4. Setting

The treatment was administered to the participants at the following locations: All participants underwent their initial therapy session at Cannectd Wellness: 712 East Palmetto Park Road, Boca Raton, FL 33432, USA.

The participants in the psychedelic group were administered intramuscular ketamine injections at Spine and Wellness Centers of America. Locations of Spine and Wellness Centers of America include: 1928 Tyler Street, Hollywood, FL 33020; 3661 S. Miami Ave, Mercy Hospital, Miami, FL, 33133; 8740 N. Kendall Drive, Suite 206–209, Miami, FL, 33176. They then completed their subsequent therapy sessions at Cannectd Wellness 24 h later.

The participants in the psycholytic group were administered oral lozenges of ketamine that they absorbed sublingually during their therapy sessions at Cannectd wellness.

Participants completed their outcome measures via the REDCap software online at the appropriate time points. Online survey administration protected participants' confidentiality and was more convenient for both participants and researchers.

## 3. Results

### 3.1. Safety and tolerability

Both ketamine-assisted psychotherapy treatment approaches were well tolerated for all participants (*N* = 10). There was no evidence of harm; no drug-related serious adverse events or clinically significant increases in blood pressure or pulse were observed.

### 3.2. Outcome measure that establishes difference between psychedelic and psycholytic treatment

#### 3.2.1. Mystical Experience Questionnaire (MEQ30)

**Figure 1:**
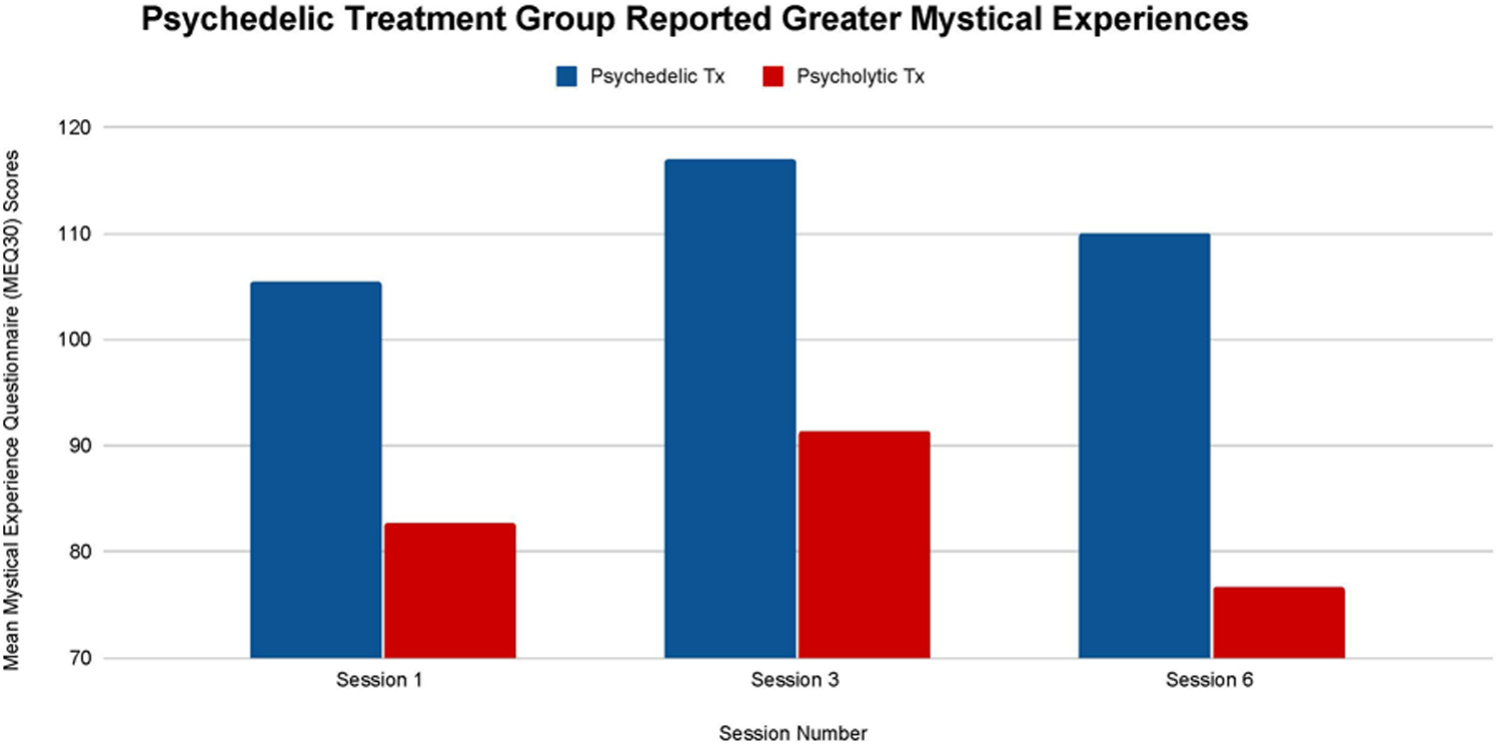
Average Mystical Experience Questionnaire (MEQ30) scores after sessions 1, 3, and 6 in both the psychedelic ketamine and the psycholytic ketamine treated groups.

The mean differences in MEQ30 scores between the psychedelic and the psycholytic group are not statistically significant after participants' first (T-1: *p *= .46), third (T-2: *p *= .46) or sixth sessions (T-3: *p = .19*). Despite limited statistical significance, results do indicate that the participants in the psychedelic treatment group had greater mean scores on the MEQ30 than the participants in the psycholytic treatment group (see Figure 1). Individuals who underwent psychedelic ketamine treatment consistently scored higher on the MEQ30 across all timepoints. The psychedelic group scored higher than the psycholytic group by 22.8 points after their first treatment session (T-1), by 25.6 points after their third treatment session (T-2), and by 33.4 points after their sixth treatment session (T-3).

### 3.3. Primary outcome measures

#### 3.3.1. Brief Pain Inventory (BPI) short form

##### 3.3.1.1. Pain Severity

**Figure 2:**
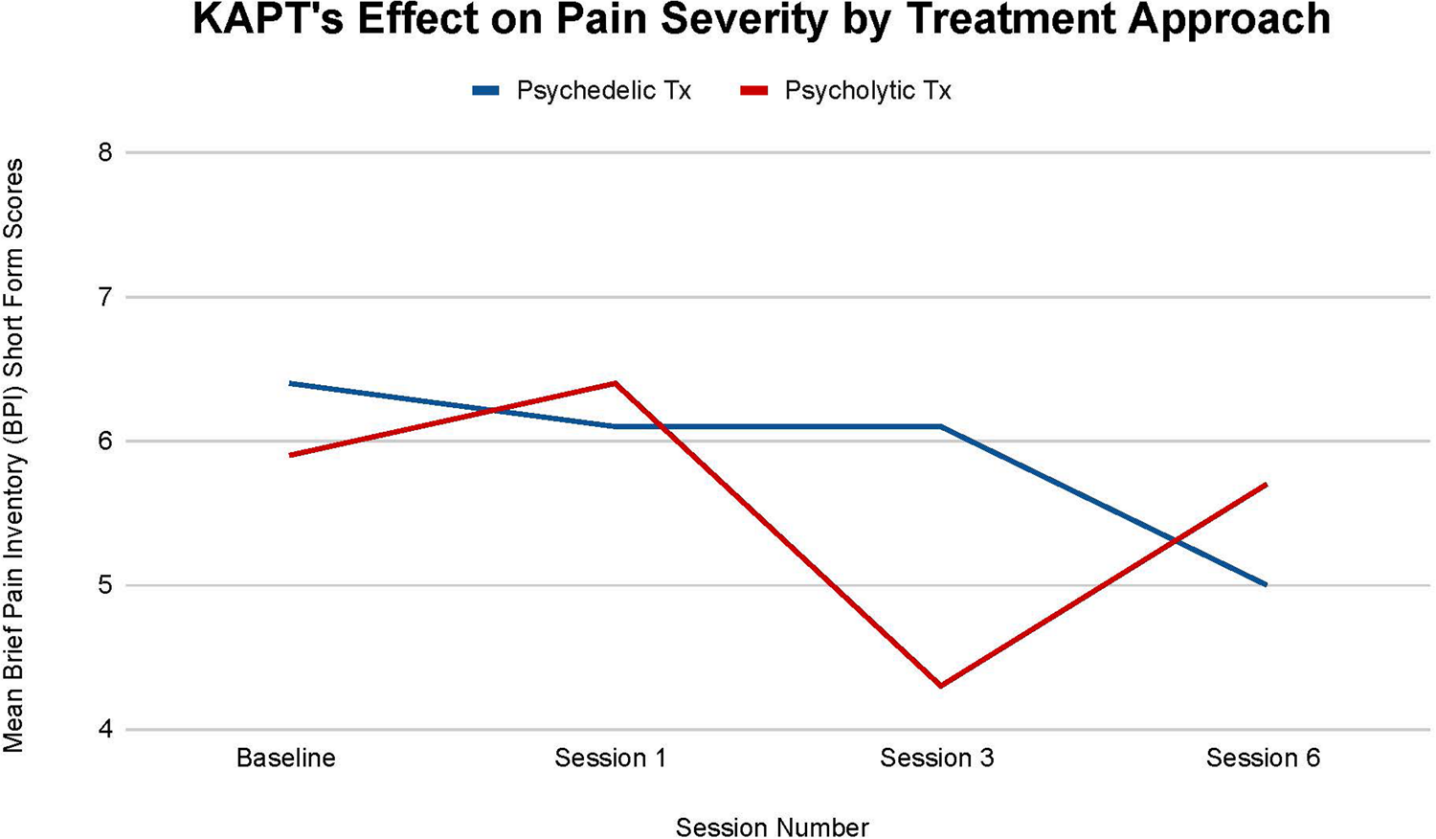
Average changes in pain severity measured through Brief Pain Inventory (BPI) short form scores at 4 timepoints (baseline and after sessions 1, 3, and 6) in both the psychedelic ketamine and the psycholytic ketamine treated groups.

Figure 2 illustrates each treatment's effect on participants' pain severity. Although no statistically significant difference is observed between each treatment's impact on participants' pain severity at all time points (T-1, *p *= .85), (T-2, *p = .34*), (T-3, *p *= .67), the psychedelic group saw a larger overall mean decrease throughout treatment. The psychedelic group's mean pain severity decreased by 21.88% from baseline (T-0) to treatment termination (T-3), while the psycholytic group's mean pain severity decreased by 3.39%.

Furthermore, the psychedelic group saw a steady mean decrease in pain severity over time with a halt at the third session (T-2). There was a 4.69% decrease between baseline (T-0) and session one (T-1), no change between session one (T-1) and session three (T-2), and a 18.03% decrease between session three (T-2) and session six (T-3). On the other hand, the psycholytic group's progression was inconsistent; the group's mean pain severity *increased* by 7.81% between baseline (T-0) and session 1 (T-1), decreased by 32.81% between session 1 (T-1) and session 3 (T-2), and then *increased again* by 24.56% between session 3 (T-2) and session 6 (T-3) (Figure 2).

##### 3.3.1.2. Pain Interference

**Figure 3:**
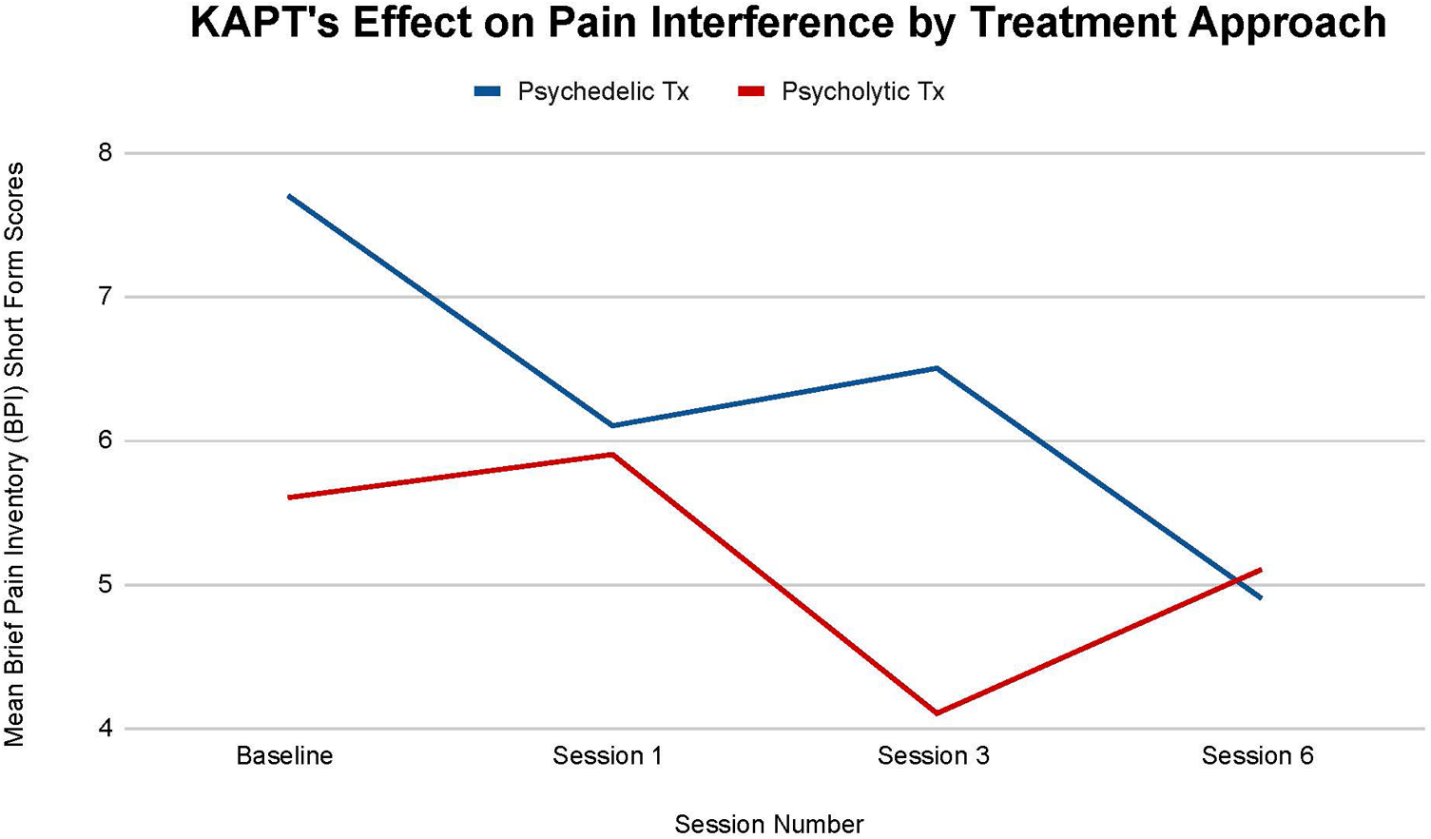
Average changes in pain interference measured through Brief Pain Inventory (BPI) short form scores at 4 timepoints (baseline and after sessions 1, 3, and 6) in both the psychedelic ketamine and the psycholytic ketamine treated groups.

Figure 3 illustrates each treatments' effect on participants' mean pain interference. Similarly to pain severity, there is no statistical significance between each treatment's impact on participants' mean pain interference at all time points: (T-1, *p *= .90), (T-2, *p = .22*), (T-3, *p *= .96). Nonetheless, small differences between each treatment are still recognized.

First, the psychedelic group saw a larger overall mean decrease in pain interference. The psychedelic group's mean pain interference decreased by 36.36%, while the psycholytic group's main pain interference decreased by 8.9% by the final treatment session (T-3) (Figure 3). Second, the mean decrease seen in the psychedelic vs. the psycholytic treatment group followed different trends. The psychedelic group's mean pain interference steadily decreased over time, despite the slight increase seen after the third session: 20.78% decrease between baseline (T-0) and session 1 (T-1), 6.15% increase between session 1 (T-1) and session 3 (T-2), 24.62% decrease between session 3 (T-2) and session 6 (T-3). Contrarily, the mean pain interference scores were inconsistent throughout treatment for the psycholytic group: scores *increased* by 5.08% between (T-0) and (T-1), decreased by 30.51% between (T-1) and (T-2), and *increased* again by 19.61% between (T-2) and (T-3) (Figure 3).

#### 3.3.2. Beck Depression Inventory (BDI)

**Figure 4:**
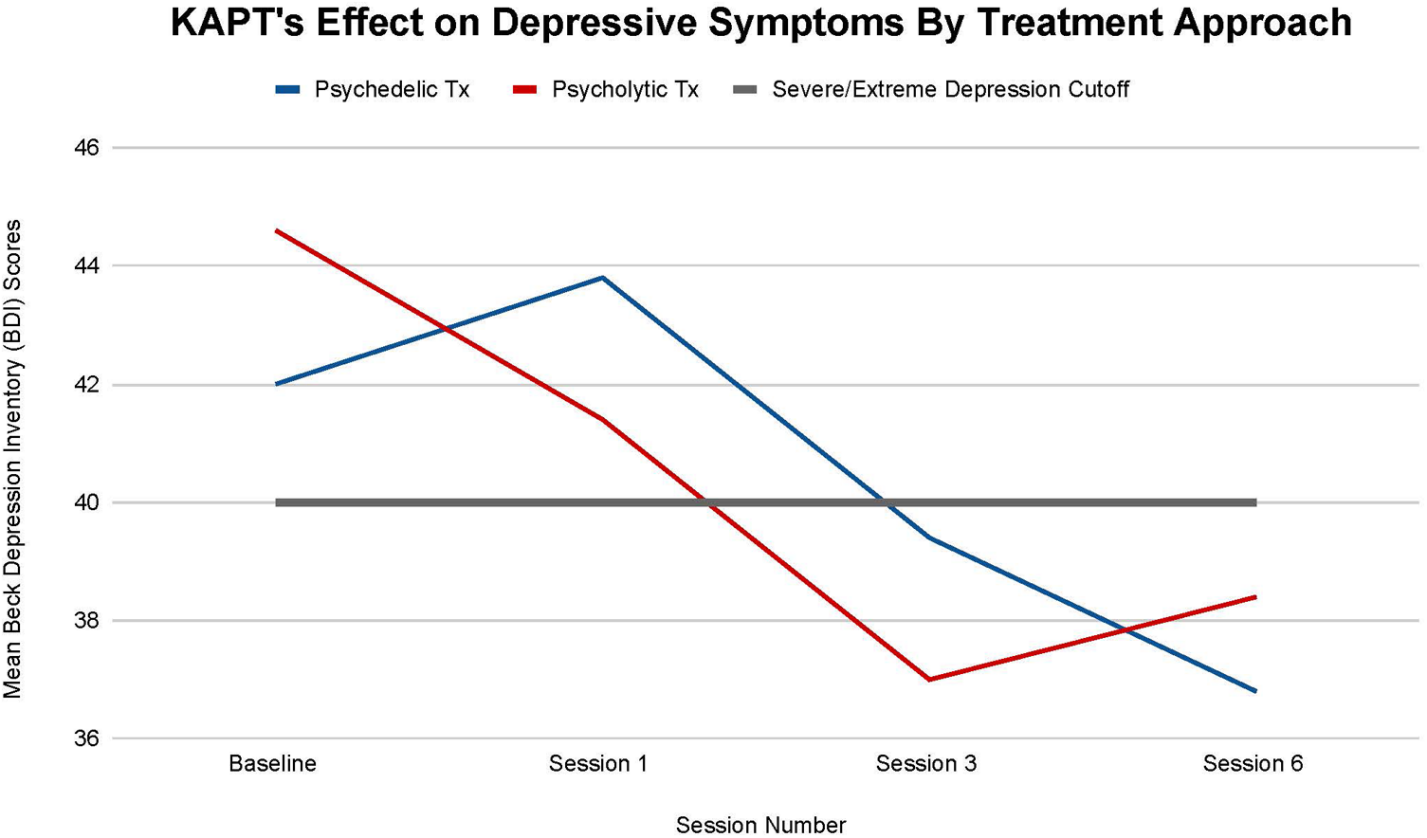
Average changes in total Beck Depression Inventory (BDI) scores at 4 timepoints (baseline and after sessions 1, 3, and 6) in both the psychedelic ketamine and the psycholytic ketamine treated groups.

All participants, regardless of treatment received, moved to a lower category of depression by the study's termination (see Figure 4). The participants in the psychedelic group enrolled in the study with a mean BDI score of 42 and left the study with a mean BDI score of 36.8. The participants in the psycholytic group enrolled in the study with a mean BDI score of 44.6 and left the study with a mean BDI score of 38.4. According to the BDI, individuals have severe depression if they score above 40 and individuals have extreme depression if they score below 40. All participants transitioned from severe to extreme after their third treatment session (T-2); psychedelic group scores declined from a mean of 43.8 to a mean of 39.4 and psycholytic group scores declined from a mean of 41.4 to 37.0. Thus, both psychedelic and psycholytic treatment approaches are associated with participants' improvement in depression severity; their decrease in symptoms moved their depression status from severe to extreme.

The differences between the psychedelic and the psycholytic group are not statistically significant at any time point. After session 1 (T-1), *p *= .83, after session 3 (T-2), *p = *.79, and after session 6, *p = *.79; *p > .*05 at all timepoints.

Despite the lack of statistically significant differences between the two treatment approaches, differences in the direction of recovery between the participants who underwent each treatment approach are worth noting. After their first treatment session (T-1), the psychedelic treatment group's symptoms *increased* by a mean of 4.12%, while the psycholytic treatment groups' symptoms *decreased* by a mean of 7.17%. After the third treatment session (T-2), both groups saw an approximately 10% mean decrease in symptoms. Between their third (T-2) and sixth (T-3) treatment sessions, the psychedelic groups’ symptoms continued to *decrease* by a mean of 6.60%, while the psycholytic group's symptoms began to *increase* by a mean of 3.70%. Differences between the two groups appeared after session 1, when the scores of participants in the psychedelic treatment group temporarily increased, and between session 3 and session 6, when the scores of participants in the psycholytic treatment group steadily increased (Figure 4).

### 3.4. Secondary outcome measures

#### 3.4.1. Generalized Anxiety Disorder (GAD-7) scale

There are no statistically significant differences in anxiety improvement seen between the two different KAPT treatment approaches at all timepoints: (T-1, *p *= .59), (T-2, *p *= .97), (T-3, *p *= .72). Nonetheless, differences between the two groups were still observed.

**Figure 5:**
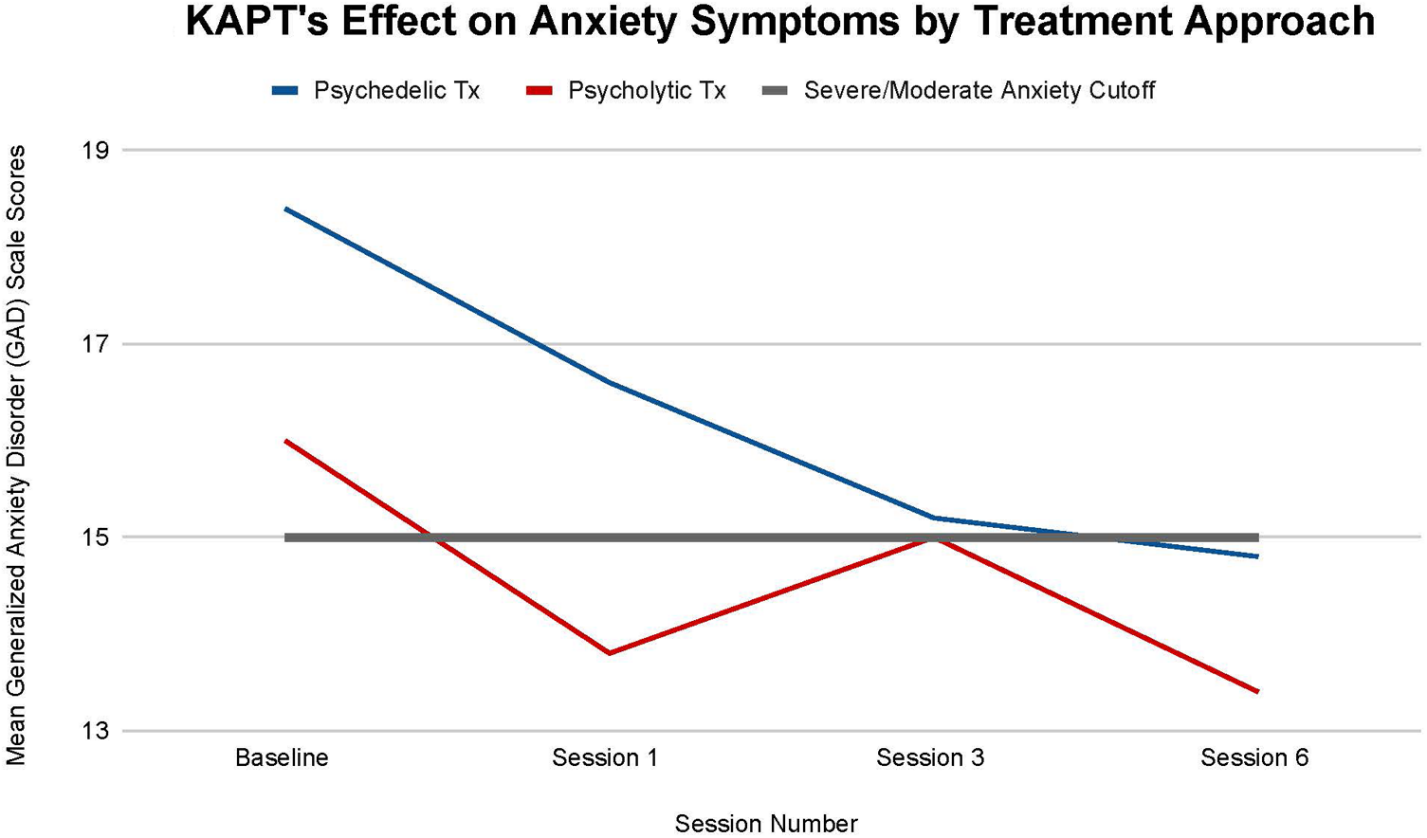
Average changes in Generalized Anxiety Disorder (GAD-7) scale scores at 4 timepoints (baseline and after sessions 1, 3, and 6) in both the psychedelic ketamine and the psycholytic ketamine treated groups.

All ten participants transitioned from having a severe GAD diagnosis at baseline (mean scores above 15 points) to having a moderate GAD diagnosis by treatment termination (mean scores below 15 points). A larger overall decrease in symptoms was seen in the psychedelic group, whose mean symptoms decreased by 19.57% by the study's completion (T-3) than in the psycholytic group, whose mean symptoms decreased by 16.25% by the study's completion (T-3). Furthermore, the psychedelic group's mean symptoms consistently declined throughout the course of treatment (T-1 = 9.78% decrease, T-2 = 8.43% decrease, T-3 = 2.64% decrease). The psycholytic group's mean symptoms, on the other hand, increased after the third treatment session (T-1 = 13.57% decrease, T-2 = 8% *increase*, T-3 = 10.67% decrease) (Figure 5).

#### 3.4.2. Post-traumatic Stress Disorder Checklist (PCL-5)

**Figure 6:**
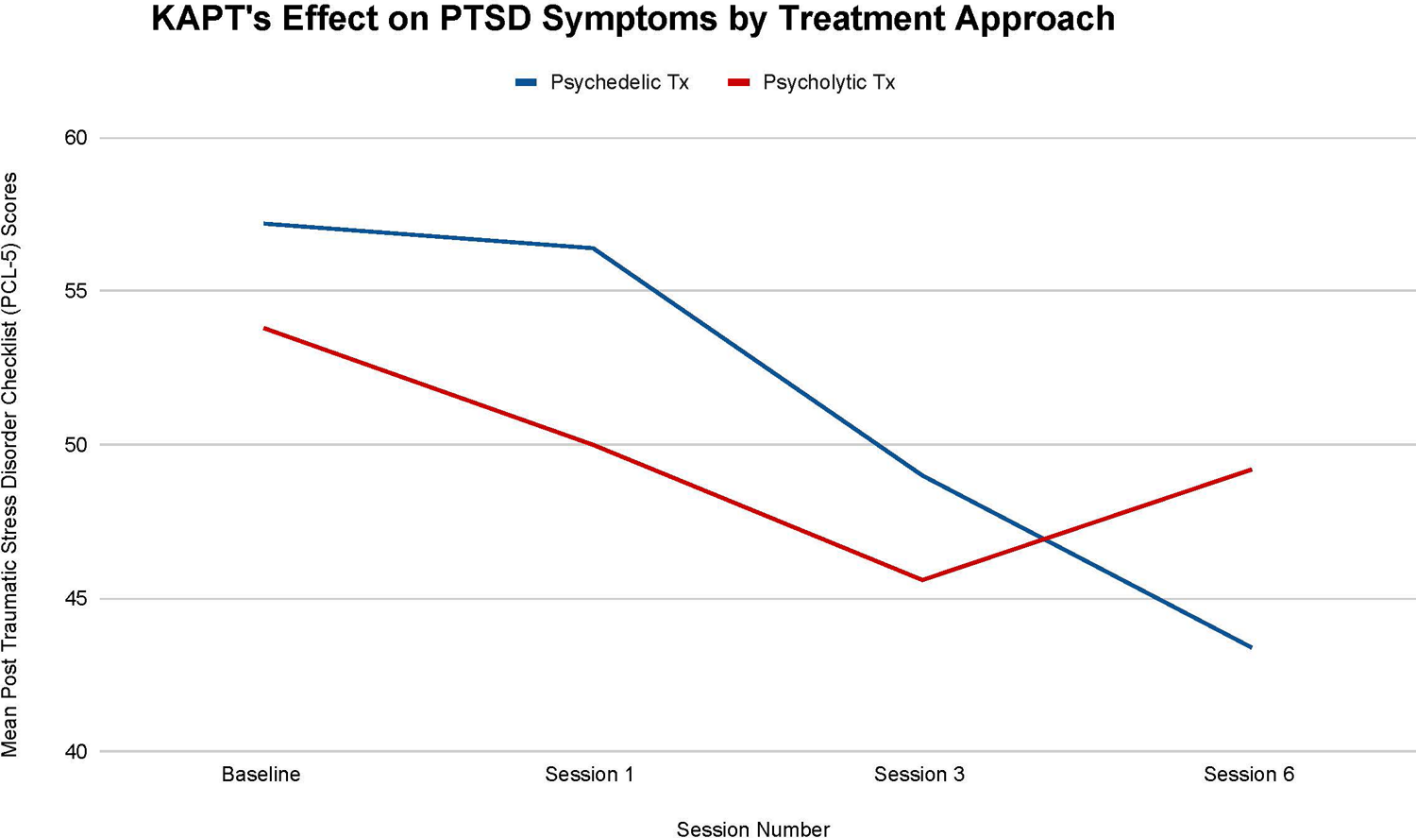
Average changes in Post-Traumatic Stress Disorder Checklist (PCL-5) scores at 4 timepoints (baseline and after sessions 1, 3, and 6) in both the psychedelic ketamine and the psycholytic ketamine treated groups.

Figure 6 illustrates that both KAPT treatment approaches reduced all participants' PTSD severity from before they received any treatment (T-0) to after they completed all 6 treatment sessions (T-3).The psychedelic group's mean PTSD score decreased by 13.8 points from baseline (T-0: 57.2 mean score) to session six (T-3: 43.4 mean score) and the psycholytic group's mean PTSD score decreased by 4.6 points from baseline (T-0: 53.8 mean score) to session six (T-3: 49.2 mean score).

There are no statistically significant differences between each treatment approach's effect on PTSD symptoms (T-1, *p *= .72; T-2, *p *= .81; T-3, *p = *.67), but the differences observed are still worth noting. The 13.8 point change seen in the psychedelic group's mean symptoms throughout treatment represents clinically significant change, whereas the 4.6 point change seen in the psycholytic group's mean symptoms represents reliable change [according to the PCL-5, a 5–10 point change is reliable (i.e., not due to chance) and a 10–20 point change is clinically significant] (33). There is a larger overall decline seen in the psychedelic group's mean PTSD symptoms, which decreased by 24.13%, than in the psycholytic group's mean symptoms, which decreased by 8.56%. Furthermore, the psychedelic group's mean PTSD symptoms continued to decrease throughout the course of treatment: there was a 40% mean decrease between T-0 and T-1, a 13.12% mean decrease between T-1 and T-2, and a 11.43% mean decrease between T-2 and T-3. On the other hand, although the psycholytic group's mean PTSD scores steadily decreased between T-0 and T-1 (by 7.06%) and between T-1 and T-2 (by 8.80%), their mean scores *increased* between T-2 and T-3 (by 7.32%) (Figure 6).

## 4. Discussion

This preliminary study investigated the relationship between two different ketamine-assisted psychotherapy approaches and symptom improvement in patients diagnosed with major depressive disorder (MDD) and a comorbid chronic pain disorder. All participants enrolled improved on all measures of symptom severity by the end of treatment. The participants who received higher doses of ketamine intramuscularly 24 h prior to therapy through the psychedelic approach saw a more drastic and consistent improvement than the participants who received lower doses of ketamine sublingually via oral lozenges during therapy through the psycholytic approach.

No statistically significant differences between scores in each treatment group were observed across all outcome measures. Nonetheless, the small sample size's lack of statistical power led researchers to still recognize clear changes and discrepancies because they may project potential, more significant results if the sample size were to increase. Implications of current findings are discussed below.

### 4.1. Mystical experiences

It can be inferred that there is a difference in the presence and intensity of mystical experiences induced by the two different ketamine treatment approaches. The psychedelic group participants experienced greater mean altered states of consciousness than the psycholytic group participants (see Figure 1). Furthermore, the low rate of mystical experiences that the psycholytic group participants did have are likely due to the placebo effect; doses administered were not potent enough to activate significant altered states.

The difference in the mystical experience that each approach catalyzed forms the framework behind this study's design. Researchers aimed to gain further insight into whether altered states of consciousness mediate ketamine's therapeutic effect. The remainder of the outcome measures administered gathered the information necessary to determine whether this difference produced distinct outcomes on participant's symptomatology. Any differences in the direction of participants' depression, pain, anxiety, and PTSD severity between each treatment group can be associated with the different levels of altered states induced.

### 4.2. Pain

Findings support KAPT's efficacy for chronic pain treatment and suggest that the psychedelic approach may be optimal. A larger decrease in both pain severity and interference scores was seen in the psychedelic treatment group (see Figures 2, 3). The psychedelic treatment group had a larger reduction in pain severity than the psycholytic treatment group by approximately 18%, and a larger reduction in pain interference by approximately 75%.

The inconsistency seen in the psycholytic group's mean pain severity and interference throughout the course of treatment further points to the favorability of the psychedelic treatment approach. The psycholytic group's regression in pain severity and interference may be due to the dose not being high enough to transmute therapeutic effects.

### 4.3. Depression

Findings suggest that ketamine assisted psychotherapy, despite the dose and route of administration, shows promise for ameliorating depressive symptomatology in this patient population. According to the criteria of the BDI, all participants, on average, transitioned from having a severe to an extreme depression categorization by the end of treatment (see Figure 4). Furthermore, the fact that participants transitioned depression categories after their third session further supports the ketamine-assisted psychotherapy standard protocol that entails not one, but multiple treatment sessions.

Although the difference in depression categorization from severe to extreme depression initially seems insignificant, it is common for people diagnosed with major depressive disorder (MDD) to only see improvement after years and sometimes decades; trial and error with different antidepressant medications and therapeutic modalities can be a lengthy, frustrating, and sometimes unsuccessful process (1, 2, 7). Therefore, although seemingly trivial, the slight improvement in depression symptoms and categorization in such a short amount of time (6 sessions/6 weeks) makes the treatment modality worth acknowledging and further exploring.

Results also support the superiority of the psychedelic treatment approach. The *increase* in depressive symptoms of the participants who received the psychedelic treatment after session 1 is interpreted as positive. This temporary, yet sharp increase in symptom severity is a crucial step of the therapeutic process; upsetting thoughts and feelings that need to be addressed tend to arise, but once felt and worked through, the depressive symptoms begin to decrease. By the end of treatment (T-3), it is evident that the increase in symptoms at (T-1) was short-term as participants' depressive symptoms continued to decline. Furthermore, whereas the depressive symptoms of the participants who received the psychedelic treatment continued to steadily decrease after the third session, the depressive symptoms of the participants in the psycholytic group increased after the third session. The difference in depression symptom direction between the two groups at this time point (T-2–T-3) further points to the psychedelic treatment approach's optimality.

There are many possible explanations for the psycholytic treatment group's regression after session 3. Their decline in improvement may be due to the dose not being strong enough to endure, to the dose not being high enough to catalyze altered states of consciousness that trigger deep insights/spiritual experiences, and/or to fears regarding treatment termination.

### 4.4. Anxiety

Findings suggest that KAPT may not only be useful for the reduction of anxiety symptoms seen in depression/chronic pain patients, but also may be an efficacious treatment for generalized anxiety disorder (GAD). Figure 5 illustrates that all participants' anxiety, whether they were in the psychedelic or the psycholytic treatment group, decreased by their final treatment session. Furthermore, all participants transitioned from a severe to moderate GAD diagnosis.

The psychedelic treatment group's larger mean decrease in anxiety symptoms throughout the course of treatment may suggest that it is the optimal treatment approach for this indication. Moreover, the psycholytic treatment group's increase in mean symptoms after session three further supports the superiority of the psychedelic treatment approach.

### 4.5. Post-traumatic stress disorder (PTSD)

Results support KAPT's efficacy for (a) reducing PTSD severity in patients diagnosed with major depressive disorder (MDD) and a comorbid chronic pain disorder and (b) for the treatment of PTSD in general. Reductions in PTSD symptoms were seen across all participants included in this study (see Figure 6).

Results imply that the psychedelic approach may be superior for this indication. First, the psychedelic treatment is associated with a larger overall mean decrease in PTSD severity than the psycholytic treatment. The mean decrease seen in the psychedelic treatment is clinically significant, whereas the mean decrease seen in the psycholytic treatment is reliable but *not* clinically significant. Second, the psychedelic group's mean symptoms decreased continuously throughout the treatment, whereas the psycholytic group's mean symptoms increased between their third and sixth treatment sessions.

Furthermore, findings suggest that there may be a connection between trauma, chronic pain, and depression. All participants enrolled in the study who had diagnoses of MDD and a comorbid chronic pain disorder *also* presented with high PTSD severity. According to the PCL-5, a total score of 31–33 or higher suggests that an individual suffers from PTSD and would benefit from treatment (33). All participants had PCL-5 scores of 50 or higher at baseline, which lowered alongside pain, depression, and anxiety severity by treatment termination. Thus, this study suggests that trauma may play a role in the development and severity of pain and depression, and treatments that target the processing of that trauma may be beneficial.

### 4.6. Limitations and suggestions for further research

This pilot study provides researchers with a basis for larger trials. The study's small sample size greatly limited the statistical power of the results. Due to economic and logistical factors, researchers only gathered data from ten participants: five participants who received psychedelic ketamine treatment and five participants who received psycholytic ketamine treatment. Due to participant variability, these findings are inconclusive and prohibit the researchers from making valid claims about the ketamine-assisted psychotherapy treatment approaches studied. Researchers must repeat this study with more participants in order to obtain results that (a) have statistical power and (b) are generalizable.

This study did not randomize participants to each treatment group because of ethical and logistical considerations. In the ketamine practice observed, clinicians determine which treatment approach better fits each of their patients' specific needs. Researchers merely collected data from individuals who were already receiving ketamine-assisted psychotherapy with each treatment approach as their standard of care. Thus, researchers can only utilize this data to make correlations/associations between each treatment approach and any symptom improvement that resulted. The treatment placement method also likely introduced bias into the study. Further research studies must incorporate randomization into their designs to eliminate bias and determine causation between each treatment and symptom alleviation.

Researchers were not able to separately investigate ketamine's dose and route of administration because of the observational nature of the study. In the ketamine practice observed, the standard psychedelic treatment entails a high dose of ketamine administered via the intramuscular route, while the standard psycholytic treatment entails a low dose of ketamine administered via the oral/sublingual route. Thus, it remains unclear whether this study's findings are associated with the different doses or the different routes of administration. Future study designs should compare different doses of ketamine with a standardized route of administration and vice versa to clearly indicate the role of each.

Because the study was observational, researchers were not able to implement a control group. Thus, whether the results were due to ketamine or due to therapy remains unclear. Future studies should incorporate control groups into their designs (i.e., one group receives ketamine alone, one group receives ketamine with therapy, one group receives therapy without ketamine) to rule out the alternative explanation that progress was solely due to therapy.

This study only utilized qualitative methods to explore the efficacy of two different KAPT treatment approaches for chronic pain/MDD comorbidity. Future studies should also incorporate qualitative methods into their study designs to investigate patient experiences with each approach.

It is important for future studies to investigate each treatment's long-term effects (i.e., 2 months, 6 months, 1-year post-treatment). Whether or not symptom improvement sustains over time is an important question that informs the favorability of each approach. It is possible that it may take time for participants to see a significant decline in symptoms due to the integrative component of ketamine-assisted psychotherapy. Greater improvement may actually be seen weeks/months after treatment, once participants process their experience and make any necessary lifestyle adjustments. Because this study's data collection period terminated upon each participant's sixth and final treatment session, it is unclear whether the mean increase in symptoms seen towards the end of the psycholytic group's course of treatment was temporary and whether the mean decrease in symptoms seen in the psychedelic group was sustainable.

Future research should investigate why the mean symptoms of the participants in the psycholytic group consistently regressed by the final treatment session across all outcome measures. It is important to gain a deeper knowledge of the reasons why this approach is associated with increases in symptoms to either (a) understand how to optimize the treatment model and reverse that effect or (b) determine that the approach is not optimal for this patient population.

Future studies should investigate ketamine-assisted psychotherapy's potential for the treatment of generalized anxiety disorder (GAD) and post-traumatic stress disorder (PTSD). Due to the improvement seen on the GAD-7 scale and PCL-5 across all participants, this pilot study suggests that KAPT shows promise for those indications.

Further research is also needed to explore the role that trauma may play in the development and maintenance of chronic pain and depressive disorders. PTSD, depression, and chronic pain have a high comorbidity and Figures 2–4, 6 illustrate that ketamine reduces all of their symptoms. Because one of ketamine's mechanisms of action is likely psychological and involves trauma processing, it seems plausible that trauma may trigger the onset of chronic pain and depression. If future studies determine that trauma is implicated in chronic pain and depressive disorders, more treatments that target the psychological healing of trauma, like ketamine, may become more readily recommended and prescribed by physicians—and thus more accessible to patients.

## 5. Conclusion

Depression and chronic pain diagnoses in the US are at an all-time high. Ketamine may be an efficacious treatment that can curb escalating rates, but limited research acts as a barrier to implementation. Clinicians need more information to embrace the novel treatment and optimize outcomes. The purpose of this study was to further understand the efficacy of different ketamine treatment approaches for chronic pain and depression comorbidity.

Findings suggest that ketamine-assisted psychotherapy may be effective for the specific population of individuals suffering from chronic pain/MDD comorbidity, as well as anxiety and PTSD. Results imply that the psychedelic approach, which utilizes the intramuscular route to deliver large doses of ketamine 24 h prior to therapy, may be more effective than the psycholytic approach, which utilizes the oral/sublingual route to deliver small doses of ketamine during therapy.

Future research must further investigate this study's implications and address its limitations. Long-term trials with larger and more diverse sample sizes, randomization, and controls for route of administration/dose are necessary to make statistically significant and generalizable claims about this novel indication. Researchers should incorporate qualitative methods into future study designs to further explore patient experiences with each treatment approach. Studies should also investigate the symptom regression seen at the end of treatment in the psycholytic group and explore KAPT's efficacy for GAD and PTSD.

On a larger note, more extensive research can pave the way towards increased administration of and access to ketamine-assisted psychotherapy treatment for this specific patient population. A greater understanding of ketamine's psychological mechanisms that reduce pain symptoms may make the redefinition of chronic pain, the subscription to a biopsychosocial model of pain by more clinicians, and the utilization of trauma-informed treatment modalities that better target the emotional roots of pain more likely. The efficacy and increased implementation of ketamine-assisted psychotherapy can also make space for the research, acceptance and utilization of other highly stigmatized psychedelic-assisted therapies that have similar effects and mechanisms of action.

## Data Availability

The raw data supporting the conclusions of this article will be made available by the authors, without undue reservation.
